# Sintilimab Combined with Lenvatinib for Advanced Intrahepatic Cholangiocarcinoma in Second-Line Setting—A Multi-Center Observational Study

**DOI:** 10.3389/fonc.2022.907055

**Published:** 2022-07-14

**Authors:** Xiaoyan Ding, Guangxin Li, Wei Sun, Yanjun Shen, Ying Teng, Yawen Xu, Wendong Li, Mei Liu, Jinglong Chen

**Affiliations:** ^1^ Department of Cancer Center, Beijing Ditan Hospital, Capital Medical University, Beijing, China; ^2^ Radiation Oncology, Beijing Tsinghua Changgeng Hospital, School of Clinical Medicine, Tsinghua University, Beijing, China; ^3^ Department of Oncology, Beijing You’an Hospital, Capital Medical University, Beijing, China

**Keywords:** intrahepatic cholangiocarcinoma, sintilimab, lenvatinib, second-line treatment, PD-L1

## Abstract

**Background:**

Patients with advanced intrahepatic cholangiocarcinoma (iCCA) have a poor prognosis and a substantial unmet clinical need. The study was aimed to investigate the efficacy and safety of sintilimab combined with lenvatinib for advanced iCCA in second-line setting.

**Methods:**

The patients at multiple centers, who progressed after the first-line chemotherapy or could not tolerate chemotherapy, were treated with the combination of sintilimab plus lenvatinib. The primary endpoint was time to progression (TTP), and the secondary endpoints included tumor objective response rate (ORR), disease control rate (DCR), overall survival (OS), and toxicity. Prognostic factors were analyzed using Cox regression analysis.

**Results:**

A total of 41 patients with advanced iCCA were enrolled for this multi-center observational study. Under a median follow-up of 12.1 months, the median age was 59 years (range, 33–75 years). Sixteen patients died of disease progression, with a median TTP of 6.6 months (95% CI, 4.9–8.3). ORR and DCR were 46.3% and 70.3%, respectively. The patients with PD-L1 TPS ≥10% reported a significantly higher ORR compared to those with PD-L1 TPS <10%, 93.8% (15/16) vs. 16.0% (4/25), *p*<0.001. The median TTP was significantly improved in patients with PD-L1 TPS ≥10%, 16.9 months (95% CI, 7.5–26.3) vs. 4.1 months (95% CI, 1.8–6.4), *p*=0.001. Attaining treatment response predicts favorable TTP in a multivariate Cox model. Treatment-emergent adverse events occurred with 70.3% probability, and no treatment-related death had been reported.

**Conclusion:**

The combination of sintilimab plus lenvatinib is effective and well tolerated for advanced iCCA in the second-line setting. PD-L1 TPS expression may predict the efficacy of the combination therapy. Further investigation is warranted to investigate this combination regimen in advanced iCCA.

## Introduction

Cholangiocarcinomas (CCAs) are highly lethal cancers arising in the epithelium of the bile duct and are typically classified as either intra- or extrahepatic cholangiocarcinoma. Intrahepatic cholangiocarcinoma (iCCA) is rare but increasing in incidence and mortality with a median overall survival (OS) of <1 year for metastatic iCCA (1). Surgical resection is the standard radical therapy. Unfortunately, only 10% of cases are eligible for surgical resection at primary diagnosis, and up to 70% of patients will relapse after radical surgery (2). Gemcitabine combined with cisplatin (GP) or 5-fluorouracil as the “gold standard” for first-line treatment has been confirmed to improve survival. However, median progression-free survival (PFS) was only about 8 months; therefore, many patients need further therapy (3, 4).

Several efforts have been carried out to explore effective and tolerable second-line therapeutic regimens. In the last decade, the ABC-06 study, which enrolled 72 patients with iCCA, is the only phase 3 randomized trial improving overall survival (OS) in patients with advanced biliary tract cancer (BTC) after progression on cisplatin and gemcitabine (5). However, the OS benefit is marginal. Lenvatinib [a known inhibitor of vascular endothelial growth factor (VEGF) receptors 1–3, fibroblast growth factor receptors (FGFRs) 1–4, and platelet-derived growth factor receptor alpha (PDGFR-α)] was tested for second-line treatment of BTC. A pilot study of 26 patients with BTC, including six ICC cases, revealed that lenvatinib has tolerable safety profile with a median PFS of 3.19 months and OS of 7.35 months (6), although with some antitumor activity, the objective response rate (ORR) was only 11.5% for mono-lenvatinib in the study.

Recently, the potential synergistic efficacy of anti-angiogenesis combined with immune checkpoint inhibitor has been demonstrated in cancer treatment (7). In addition, the success of the combination of immune checkpoint inhibitors, such as sintilimab with a bevacizumab biosimilar (IBI 305) (8) or pembrolizumab plus lenvatinib (9, 10), have been reported in unresectable hepatocellular carcinoma. Considering that iCCA sometimes invade liver parenchyma and adopts combined features, immune checkpoint inhibitors combined with lenvatinib may have excellent prospects in iCCA. Herein, we conducted this multi-center trial to evaluate the efficacy and safety of sintilimab plus lenvatinib in second-line setting for advanced iCCA in the real world.

## Patients and methods

### Patients

Patients with unresectable iCCA were enrolled in this study. Eligibility criteria were the following: age ≥18 years; histologically confirmed as iCCA; at least one measurable lesion according to Response Evaluation Criteria In Solid Tumors (RECIST) version 1.1 (11); metastatic or locally advanced unresectable disease documented on diagnostic imaging studies, staging II–IV according to the American Joint Committee on Cancer (AJCC) 8th Edition Cancer Staging System (12); progression after first-line chemotherapy or intolerable to first-line chemotherapy; treated with sintilimab plus lenvatinib; and locoregional therapy allowed. We excluded patients if they had central nervous system metastases, received first-line immunotherapy, received other immune checkpoint inhibitors or other target therapy, had missing data, and lost to follow-up.

The study was reviewed and approved by the ethics committee of Capital Medical University-affiliated Beijing Ditan Hospital. All study patients provided informed written consent prior to study enrollment. The ethics committee at each institution approved this study. The study protocol was conducted according to the principles of the Declaration of Helsinki.

### Study Design and Treatments

This retrospective multi-center study was conducted at three institutions in Beijing, China: Capital Medical University-affiliated Beijing Ditan Hospital [n=21], Capital Medical University-affiliated Beijing You’an Hospital [n=10], and Tsinghua Changgung Hospital [n=10].

The medical records were retrieved, and clinical data were collected regarding patients’ characteristics, clinical presentation, treatment, and clinical course. Patients who were enrolled from October 31, 2019 to October 31, 2021 received the combination of sintilimab plus lenvatinib. Treatment was continued until disease progression, drug intolerance, or withdrawal of consent from the study.

### Systemic Therapy

Patients were initiated with lenvatinib (Lenvima^®^; Eisai Co., Ltd., Tokyo, Japan) daily within 7 days of transarterial chemoembolization (TACE) or radiation therapy. Patients who weighed <60 kg were administered with lenvatinib 8 mg once daily; those who weighed ≥60 kg, 12 mg once daily; and those with a Child–Pugh grade B, 8 mg once daily, regardless of weight. Patients were administered with sintilimab at 200 mg intravenously on day 1 of a 21-day therapy cycle after the first dose of lenvatinib.

### Transarterial Chemoembolization

After puncturing the femoral artery, the portal vein patency and liver blood supply were confirmed. The patients then underwent distal super-selective 5-F catheterization of the tumor-feeding hepatic arteries with a mixture of lipiodol and Oxaliplatin. Oxaliplatin (60 mg/m^2^) was infused *via* the catheter, and iodized oil (Lipiodol Ultrafluido; Guerbet, Aulnay-sous-Bois, France) was used to embolize tumor-feeding arteries. The entire tumor burden was treated with TACE. The TACE procedure was only performed once before the systemic therapy.

### PD-L1 Expression

Tumor tissue samples for analyzing PD-L1 expression were obtained by needle biopsy or surgery. Preserved tumor specimens were formalin fixed, paraffin embedded (FFPE), and then cut into 4–5-mm-thick sections for further staining. The primary antibody used was anti-PD-L1 (IHC 22C3 pharmDx, Dako North America, Agilent Technologies). Expression was categorized by Tumor Proportion Score (TPS), which was defined as the percentage of tumor cells with membranous PD-L1 staining (13). Next-generation sequencing was carried out by OrigiMed, a College of American Pathologists-accredited and Clinical Laboratory Improvement Amendments-certified laboratory in Shanghai, China.

### Efficacy Assessment

Time to progression (TTP), defined as the time from treatment initiation until disease progression, was the primary endpoint. Secondary endpoints included OS (defined as the time from treatment initiation to death), objective response rate (ORR, including the rate of complete response plus partial response), disease control rate (DCR, including complete response, partial response, and stable disease), and adverse event (AE) rates. The efficacy evaluation was conducted according to the RECIST1.1 criteria, including complete response (CR), partial response (PR), stable disease (SD), and progressive disease (PD) (12). Tumor assessments were performed based on computed tomography and/or magnetic resonance imaging evaluation as defined by RECIST 1.1 at baseline and every 6–8 weeks thereafter. After disease progression, follow-up was performed every month.

### Statistical Analysis

All data analyses were performed using the SPSS software (version 21.0; SPSS, Chicago, IL, USA). Demographic data, outcome data, and other clinical parameters were presented as the frequency for categorical variables. Continuous variables were expressed as median (range) or mean (± SD). The ORR and DCR and corresponding 95% CIs were calculated using the Clopper–Pearson method. Differences in the outcome data of groups were compared using the Chi-square test. The Kaplan–Meier method and log-rank test were used to evaluate survival endpoints between two different subgroups. The hypotheses of ORR, overall survival, and progression-free survival were assessed by chi-squared or Fisher exact tests between different levels of PD-L1 TPS (≥10% or <10%). A non-parametric log-rank test was used to evaluate the HRs and 95% CIs estimated using a Cox proportional hazards model. All statistical analyses were two-sided, and *p*-values <0.05 were considered significant.

## Results

### Baseline Characteristics

**Figure 1:**
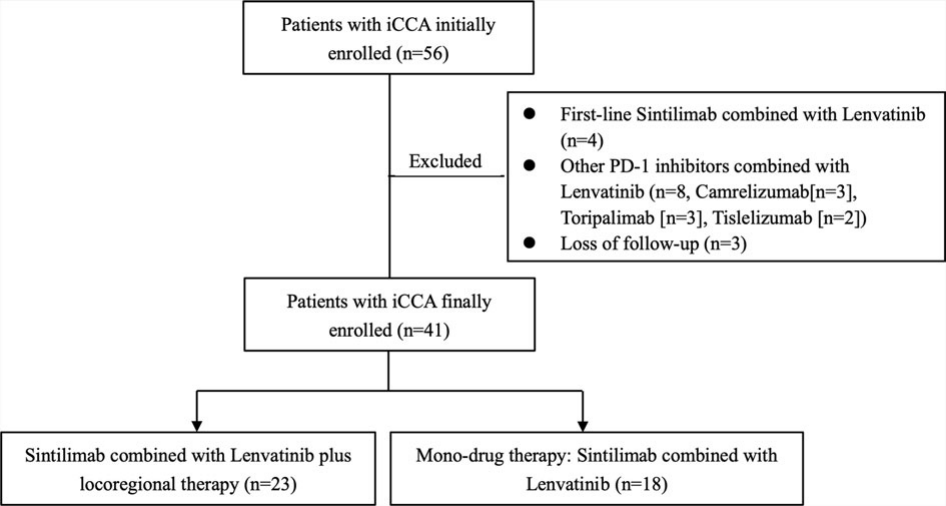
Study profile. iCCA, intrahepatic cholangiocarcinoma.

Between October 31, 2019 to October 31, 2021, 56 medical records were reviewed; 41 eligible patients were enrolled (**Figure 1**). The baseline demographics and clinical characteristics of patients are summarized in **Table 1**. The median age of the entire cohort was 59 years (range, 33–75 years), and the male/female ratio was 25:16. Most of the participants had Eastern Cooperative Oncology Group (ECOG) performance status scores of 0 and 1 (95.1%; n=39). All cases were Child–Pugh class A, with 10 cases (24.4%) suffering from liver cirrhosis. Seventeen cases were hepatitis B virus (HBV) infected. Among the 41 patients, there were 11 cases diagnosed as post-surgery metastatic ICC, and 30 cases were unresectable at primary diagnosis due to locally advanced or advanced disease (5 cases with stage II, 3 cases with stage IIIB, and 22 cases with stage IV). Thirty-two patients had lymph node metastasis (including hilar lymph nodes, retroperitoneal lymph nodes, and supraclavicular lymph nodes), and 13 patients had other organ metastasis. There were 10 patients who were intolerable to first-line chemotherapy regimen due to liver cirrhosis or gastrointestinal side effects. Thirty-one cases progressed after first-line chemotherapy. The specific regimens are depicted in **Table 1**. Seventeen patients were combined with TACE, and six patients received radiation therapy at time of systemic therapy.

**Table 1:** Baseline characteristics and demographics.

Characteristic	No. (%) (N=41)
Age, median (range, year)	59 (33–75)
Sex	
Male	25 (61.0)
Female	16 (39.0)
ECOG	
0	12 (29.3)
1	27 (65.8)
2	2 (4.9)
Hepatitis virus infection	
HBV	17 (41.5)
HCV	2 (4.9)
Cirrhosis	10 (24.4)
Disease stage	
Locally advanced	8 (19.5)
Metastatic	33 (80.5)
Metastatic site	
Lymph node	32 (78.0)
Lung	13 (31.7)
Bone	10 (24.4)
Liver	9 (22.0)
Others	5 (12.2)
Diameter of target lesion, median (cm, range)	8.6 (1.5–16.8)
Baseline serum biomarkers (median, range)	
CEA (ng/ml)	3.7 (1–185.5)
AFP (ng/ml)	4.0 (0.9–67,251)
CA19-9 (IU/ml)	67.6 (1.3–62,677)
≥100	17 (41.5)
<100	24 (58.5)
Tumor PD-L1 expression TPS, median(range)	7 (0–70)
≥10%/<10%, n (%)	16 (39.0)/25(61.0)
≥1%/<1%, n (%)	33 (80.5)/8 (19.5)
Prior curative surgery	11 (26.8)
Previous first-line chemotherapy	
Intolerable to first-line chemotherapy	10 (24.4)
S-1	8 (19.5)
Gemcitabine combined with oxaliplatin	2 (4.9)
Progressed after first-line chemotherapy	31 (75.6)
nab-paclitaxel plus gemcitabine	2 (4.9)
Gemcitabine Plus Platinum	11 (26.8)
S-1	18 (43.9)
Combination with locoregional therapy	23 (56.1)
TACE	17 (41.5)
Radiation	6 (14.6)
Next-generation sequencing, n (%)	15 (36.6)
TMB Muts/Mb; median (range)	6.3 (1.0–16.8) *

TPS, Tumor Proportion Score; nab, nanoparticle albumin-bound; *Case 6, harboring FGFR2 exon17-BICC 1 exon 3 fusion mutation with TMB 1.1 Muts/Mb; S-1, gimeracil and oteracil potassium capsule; TACE, transarterial chemoemolization.

At baseline, the median PD-L1 TPS expression was 6% (7%–70%), 16 cases (39%) were ≥10%, and 25 cases (61%) were <10%. Fifteen (36.6%) patients had done next-generation sequencing. All of the cases were microsatellite stable (MSS). The median tumor mutation burden (TMB) was 6.3 Muts/Mb (range, 1.0–16.8). Of those, two cases were categorized to be TMB-H, 14.8 and 16.8 Muts/MB, respectively. The case with TMB of 14.8 Muts/MB reported the PD-L1 TPS expression of 2%, and harbored an KRAS mutation, TP53 mutation, and c-Met amplification. The other case with TMB of 16.8 Muts/MB showed that the PD-L1 TPS was 10% and harbored TP53 and PBRM1 mutation.

### Treatment Efficacy

**Figure 2:**
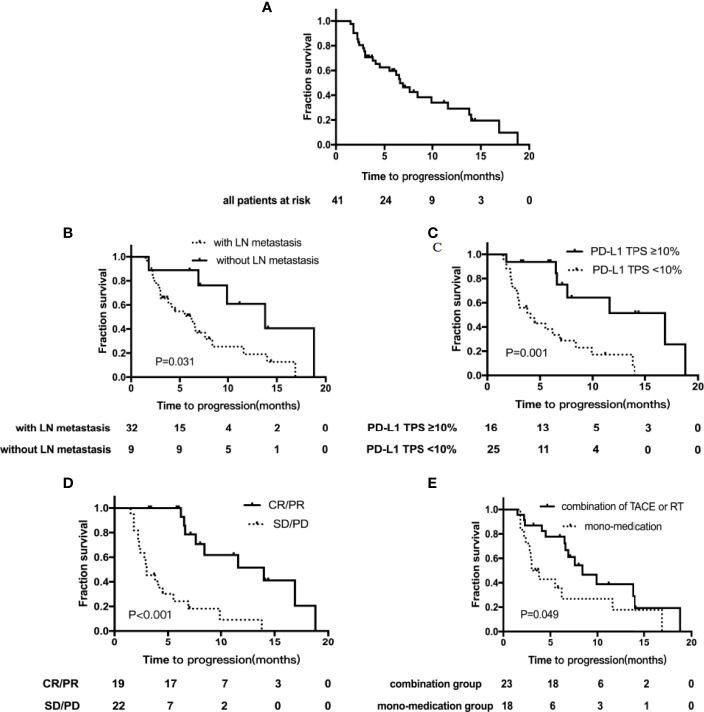
Kaplan–Meier curves showing the time to progression (TTP). **(A)** TTP for the whole group; **(B)** TTP by LN metastasis subgroups; **(C)** TTP by PD-L1 TPS expression subgroups; **(D)** TTP by tumor remission subgroups; **(E)** TTP by different treatment subgroups. TACE, transarterial chemoembolization; RT, radiation therapy; TPS, tumor proportion score; LN, lymph node.

**Figure 3:**
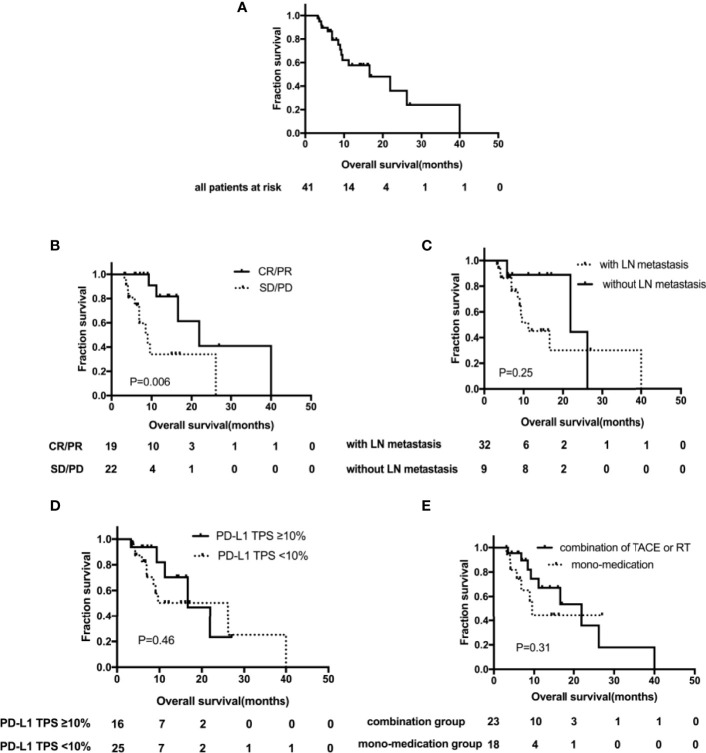
Kaplan–Meier curves showing overall survival (OS). **(A)** OS for the whole group; **(B)** OS by LN metastasis subgroups; **(C)** OS by PD-L1 TPS expression subgroups; **(D)** OS by tumor remission subgroups; **(E)** OS by different treatment subgroups. TACE, transarterial chemoembolization; RT, radiation therapy; TPS, tumor proportion score; LN, lymph node.

Till December 31, 2021, the median follow-up time was 12.1 months (5.1–19.1 months). Of the cohort, 68.3% (n=28) reported disease progression, and the median TTP was 6.6 months (95%CI, 4.9–8.3) (**Figure 2A**). The median TTP of patients with lymph node metastasis was significantly worse, 6.2 months (95% CI, 3.5–8.9) vs. 13.8 months (95% CI, 6.1–21.5), *p*=0.031 (**Figure 2B**). The median TTP was significantly prolonged in patients with baseline PD-L1 TPS ≥10% compared to those with PD-L1 TPS <10%, 16.9 months (95% CI, 7.5–26.3) vs. 4.1 months (95% CI, 1.8–6.4), *p*=0.001(**Figure 2C**). In addition, in patients who achieved tumor remission, the median TTP was significantly improved compared to those with SD or PD, 14.0 months (95% CI, 6.0–22.0) versus 3.0 months (95% CI, 1.9–4.1), *p* < 0.001 (**Figure 3D**). The addition of locoregional therapy including TACE or RT did significantly prolong the median TTP compared to the control group, 8.4 months (95% CI, 5.2–11.6) versus 3.0 months (95% CI, 1.9–4.1), *p*=0.049 (**Figure 2E**).

Oof the patients, 39.0% (n=16) died of disease progression, with a median OS of 16.6 months (95% CI, 5.0–28.2) (**Figure 3A**). Except that, the median OS of patients with tumor remission was significantly improved, 21.9 months (95% CI, 11.1–32.7) vs. 9.0 months (95% CI, 5.7–12.3), *p* = 0.006 (**Figure 3B**). OS was analyzed for several factors; in univariate analysis, no significant correlation had been identified, including lymph node metastasis vs. lymph node negative (11.2 months [95%CI, 5.1–17.3] vs. 21.9 months [95% CI, 0–44.5], *p* = 0.25) (**Figure 3C**); baseline TPS PD-L1 ≥10% <10% (16.6 months [95%CI, 6.9–26.3] vs. 26.2 months [95% CI, 6.6–45.8], *p*=0.46) (**Figure 3D**) and the combination of TACE or RT vs. non-locoregional therapy (21.9 months [95%CI, 9.9–33.9] vs. 9.6 months [95% CI, 8.0–11.2], *p*=0.31) (**Figure 3E**).

Of the entire group, 46.3% (n=19) achieved a partial response, 29.3% (n=12) had stable disease, and 24.4% (n=10) had progressive disease. The overall ORR was 46.3% (95%CI: 30.7%-62.6%), and the DCR was 75.6% (95% CI, 59.7%–87.6%). As for the two cases harboring TMB-H, one case with TMB of 14.8 Muts/MB reported PD with an PFS of 2.2 months and an OS of 6.9 months. The other case with TMB of 16.8 Muts/MB reported PR, who remained in disease remission at the last follow-up.

**Figure 4:**
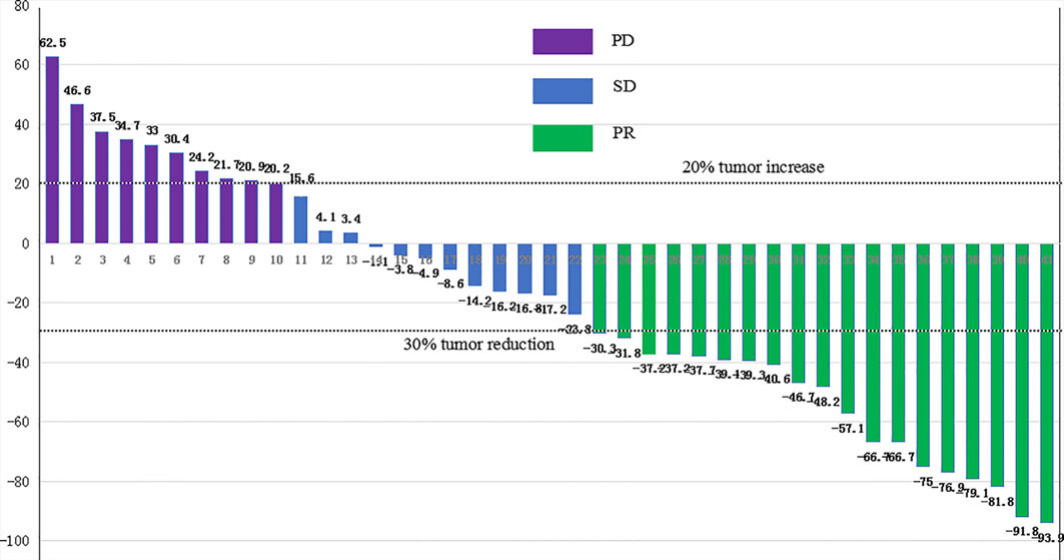
The waterfall diagram of tumor efficacy. PR, partial response; SD, stable disease; PD, progression disease.

**Figure 5:**
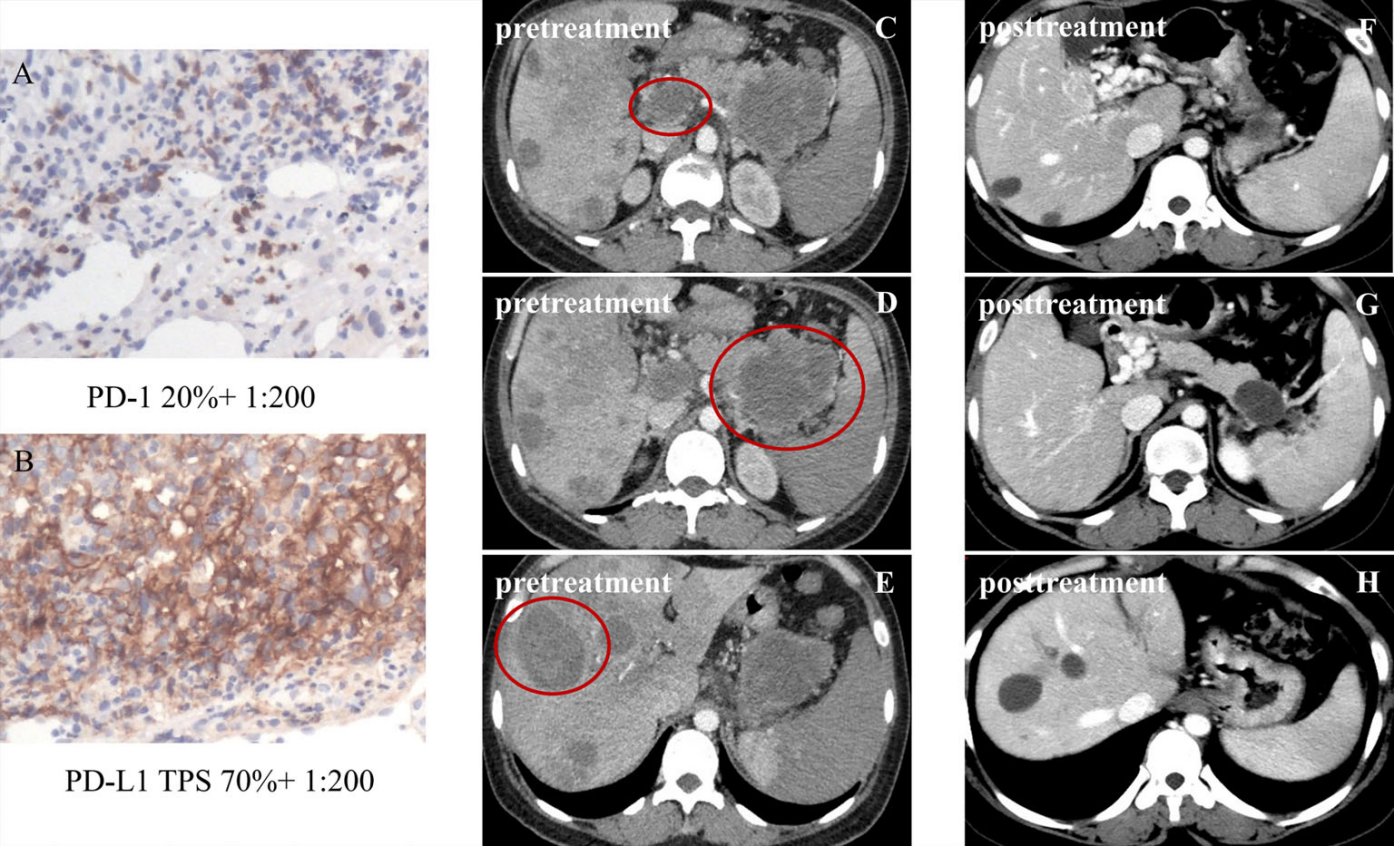
Partial response in a patient with metastatic intrahepatic cholangiocarcinoma (iCCA), after four cycles of sintilimab plus lenvatinib. The tumor was defined as PD-1 20% and PD-L1 TPS 70% **(A, B)**; pretreatment figures are depicted in panels **(C–E)**, of metastatic iCCA with lymph node metastasis and pancreatic metastasis; post-treatment figures of metastatic ICC are depicted in panels **(F–H)**. The sizes of the liver tumors, the retroperitoneal lymph node, and the metastatic pancreas obviously shrank, and some liver tumors disappeared. Until now, no disease progression had been demonstrated.

The 16 patients with baseline PD-L1 TPS ≥10% reported a significantly higher ORR compared to the cases with PD-L1 TPS <10%, 93.8% (95%CI, 69.8%–99.8%) versus 16% (95%CI, 4.5%–36.1%), *p*<0.001. The waterfall diagram of tumor efficacy is as the following **Figure 4**. One female case with PR (PD-1 +20%, PD-L1 +70%) is displayed in **Figure 5**. She had multiple organ metastasis, including the retroperitoneal lymph node, liver, and pancreatic metastasis. Till the last follow-up, no disease progression had been demonstrated, and the disease remission time was 14.1 months. Regretfully, next-generation sequencing was not taken either for tumor tissue or serum.

### Multivariable Analyses

Multivariable Cox proportional hazards analysis revealed that patients who achieved an objective response had significantly improved TTP (HR, 0.098 [95% CI, 0.019–0.49]; *p*=0.005). However, no significant prognostic factors were identified in the multivariable Cox proportional hazards model for OS. CA19-9 (IU/ml) level, ECOG performance status, the combination of TACE or RT, a maximum target tumor ≥7 cm, PD-L1 TPS ≥10%, and extrahepatic metastasis were not associated with TTP or OS (**Table 2**).

**Table 2:** Multivariable Cox proportional hazard model for TTP and OS.

Characteristic	Time to Progression					Overall Survival	
	28 events					16 events	
	Total (n)	HR	95% CI	*p*-value	HR	95% CI	*p*-value
Treatment group						
TACE or RT vs.	23	0.60	0.23–1.57	0.30	–	–	–
mono-drug group(reference)	18	1			–		
ECOG						
0 vs.	12	–	–	–	0.26	0.028–2.49	0.24
1,2 (reference)	29	–			1		
Maximum liver tumor						
≥7cm	23	–	–	–	2.96	0.72–12.16	0.13
Yes vs.	18	–			1		
No (reference)						
Sex						
Male vs.	25	–	–	–	3.12	0.59–16.52	0.18
Female (reference)	16	–			1		
PD-L1 TPS score						
≥10% vs.	16	0.62	0.12–3.18	0.56	–	–	–
<10% (reference)	25	1			–		
Lymph node metastasis						
Yes vs.	32	5.64	1.43–22.25	0.014	–	–	–
No (reference)	9	1			–		
Lung metastasis						
Yes vs.	13	–	–	–	–	–	–
No (reference)	28	–			–		
CA19-9 ≥100 IU/ml						
Yes vs.	17	–	–	–	2.41	0.67–8.68	0.18
No (reference)	24	–			1		
Efficacy						
CR or PR vs.	19	0.098	0.019–0.49	0.005	0.29	0.083–1.05	0.059
SD or PD (reference)	22	1			1		

Numbers in parentheses are the 95% confidence intervals. Objective response is defined as achieving a complete or partial response based on the Response Evaluation Criteria. 1.1 for Solid Tumors.

CI, confidence interval; CR, complete response; ECOG PS, Eastern Cooperative Oncology Group; HR, hazard ratio; OS, overall survival; PD, progressive disease; PR, partial response; SD, stable disease; TACE, transarterial chemoembolization; TTP, time to progression; TPS, tumor proportion score.

### Adverse Events

The overall incidence of treatment-related AEs of any grade was 70.3% (n=26). Most adverse events were mild to moderate (**Table 3**). The most frequently reported treatment-related AEs (≥15%) were aspartate transaminase elevation (n=15, 40.5%), alanine transaminase elevation (n=15, 40.5%), hypertension (n=13, 35.1%), fatigue (n=12, 32.4%), decreased appetite (n=11, 29.7%), diarrhea (n=10, 27.0%), increased total bilirubin (n=8, 21.6%), increased direct bilirubin (n=8, 21.6%), rash or desquamation (n=8, 21.6%), proteinuria (n=7, 18.9%), fever (n=6, 16.2%), leukocytopenia (n=6, 16.2%), and neutropenia (n=6, 16.2%). The overall incidence of ≥ grade 3 AEs was 14/37 (37.8%). Only one case was diagnosed as grade 4 immune-related hepatitis, which was relieved without sequelae after steroid treatment (with a daily dose of oral prednisone 50 mg for continuous 7 days, then with a gradual reducing dose for 3 weeks), resulting in permanent withdrawal of sintilimab. One patient was diagnosed with grade 4 elevated total bilirubin and direct bilirubin, which was attributed to disease progression. Three cases were diagnosed as grade 3 hypertension; all of those were administered with the combination of calcium channel antagonists and angiotensin-converting enzyme inhibitors. Five cases of serious adverse events (SAEs) were reported, in which four cases may be related to TACE treatment, two cases of abdominal infection, one case of acute cholecystitis, one case of liver abscess, and one case of liver failure, which may be related to radiation therapy. All of the SAEs were relieved without sequelae (**Table 3**). Dose reductions and discontinuations were reported in 27.7% (13 of 47) of the patients. No treatment-related deaths were reported.

**Table 3:** Treatment-emergent adverse events.

Treatment-emergent adverse events	Any grade, n (%)	Grade 3/4, n (%)
ALT elevation and AST elevation	15 (40.5)	1 (2.7)
Hypertension	13 (35.1)	3 (8.1)
Fatigue	12 (32.4)	0
Decreased appetite	11 (29.7)	0
Diarrhea	10 (27.0)	1 (2.7)
Bilirubin elevation	9 (24.3)	1 (2.7)
Rash or desquamation	8 (21.6)	8 (21.6)
Proteinuria	7 (18.9)	1 (2.7)
Hypothyroidism	7 (18.9)	0
Leukocytopenia and neutropenia	6 (16.2)	0
Fever	6 (16.2)	1 (2.7)
Thrombocytopenia	6 (16.2)	1 (2.7)

## Discussion

We investigated 41 cases that received sintilimab plus lenvatinib in second-line therapy for advanced iCCA; median TTP was 6.6 months, and median OS was 16.6 months. This combination regimen showed encouraging clinical benefit in real world, longer than most reported studies. The ABC-06 study reported a median PFS of 4 months (5); while lenvatinib achieved 3.2 months (6), pembrolizumab was just 2 months (14). Recently, a study reviewed nine iCCA cases treated with lenvatinib and immune checkpoint inhibitors and showed a median PFS of 8.3 months (10). Although its sample size is too small to draw a conclusion, the trend of a longer PFS is in accordance with our study. One possible explanation might be that the prognosis of iCCA tends to be better than extrahepatic cholangiocarcinoma. A *post-hoc* analysis of the ABC-01, ABC-02, and ABC-03 Clinical Trials showed that the median OS of iCCA was 15.4 months compared to 12.6 months for the whole cohort (15). Another potential explanation is the synergetic effect of PD-1 inhibitor and lenvatinib. Lenvatinib plus immune checkpoint inhibitors have been shown to specifically decrease the proportion of Treg cells, activate the immune pathways, and inhibit transforming growth factor beta (TGFβ) signaling (16). Thus, the combination showed robust antitumor activity in a phase Ib trial involving 100 patients with previously untreated unresectable HCC (10).

Patients with iCCA are more likely to have liver-only disease, which might suit locoregional treatments (17). In our study, 56.1% (n=23) received locoregional treatments. These patients had a significantly prolonged TTP compared to the mono-medication group (8.4 months [95% CI, 5.2–11.6] versus 3.0 months [95% CI, 1.9–4.1], *p*=0.049). The results suggest that locoregional therapy would increase the antitumor activity of the combination therapy of lenvatinib and sintilimab. It is in accord with a previous review about cholangiocarcinoma and combined hepatocellular carcinoma (17). However, the efficacy outcomes should be cautiously interpreted because of different tumor type, various tumor loads, and limited sample number.

Due to the complexity of the immunological microenvironment and heterogeneity of tumors, predictive biomarkers for immunotherapy are unclear. PD-L1 is a potential biomarker of immunotherapy. Our study took 10% as a cutoff of PD-L1 status. Of the 16 PD-L1-positive patients, a statistically significantly superior median TTP and ORR were observed compared to PD-L1-negative iCCAs (16.9 months versus 4.1 months; *p*= 0.001; 93.8% vs. 16.0%, *p*<0.001). However, no significant differences had been detected in OS between PD-L1 TPS ≥10% and PD-L1 TPS <10% subgroup. Many trials took 1% as a cutoff of PD-L1 status and showed discord findings. KEYNOTE-158 reported no significant differences in ORR and OS (14), so do a phase 2 study of 22 previously treated BTCs, using the combination of Apatinib plus Camrelizumab (18). Only in the Nivolumab monotherapy study, a statistically significantly superior median PFS was observed compared to PD-L1-negative BTCs (10.4 versus 2.3 months; *p* < 0.001) (19). When ≥5% of tumor cells expressing PD-L1 was defined as a cutoff, patients, treated with pembrolizumab plus lenvatinib as non-first-line therapy, with positive PD-L1 expression had a significantly higher clinical benefit rate (CBR) than patients with negative PD-L1 expression (72.7%, 8/11 vs. 23.8%, 5/21, *p*=0.021) (20). Consequently, significantly improved survival outcomes in both PFS and OS had been observed in patients with positive PD-L1 expression. Taken together, tumor PD-L1 expression might be a potential predictive biomarker for the combination of lenvatinib plus PD-1 inhibitors, which needs further investigation.

Regarding the predictive value of mismatch repair deficiency (dMMR)/high microsatellite instability (MSI-H) and TMB, few data are available so far (21). Nevertheless, it has demonstrated the landscape of molecular mutations and identified several special driver genetic alterations in BTCs; for example, ICCs have the highest of mutations in isocitrate dehydrogenase 1 (IDH1) and fibroblast growth factor receptor (FGFR) fusions that are of special interest (22). In addition, the responders in nivolumab monotherapy and pembrolizumab were microsatellite stable (MSS) (14, 19). Other potential predictive biomarker needs to be explored.

The safety profile of lenvatinib plus sintilimab was consistent with previous reports of lenvatinib plus pembrolizumab (9, 20). No treatment-related mortality was observed, but five SAEs had been reported, including four cases with infection and one case with liver failure. In addition, the most common AEs included aspartate transaminase elevation (40.5%) and alanine transaminase elevation (40.5%). The occurrence of ALT and AST elevation were higher than those in other studies in BTCs (17, 19). The reason may be that 56.1% of patients received locoregional therapy TACE or radiation therapy. The addition of locoregional therapy TACE or RT did not significantly improve OS; thus, the benefits and risks should be fully assessed.

The limitations of our study must be acknowledged, highlighting the need for well-designed prospective clinical trials with control arms to determine the efficacy and safety of this combined regimen in a second-line setting. Our present study is an investigator-initiated retrospective multi-center study with underlying selection bias. Considering the high proportion of PD-L1 overexpression (≥10%, 39.0% of all patients), it is possible that the encouraging efficacy in our cohort partly driven by these patients, since patients with high PD-L1 expression were more likely to get more benefit from an anti-PD1 inhibitor. Other confounding factors, such as the addition of TACE or RT, should also be considered in efficacy and survival analyses.

In conclusion, we preliminarily reported a combined regimen of lenvatinib plus sintilimab in patients with advanced ICCs in a second-line setting. The combination is well tolerated and showed encouraging efficacy, which might be more effective in patients with PD-L1 TPS over 10%. Further investigation of this current regimen in prospective multi-center clinical practice is warranted.

## Data Availability Statement

The original contributions presented in the study are included in the article/**Supplementary Material**. Further inquiries can be directed to the corresponding authors.

## Ethics Statement

The studies involving human participants were reviewed and approved by the institutional review board of Beijing Ditan Hospital, Capital Medical University. The patients/participants provided their written informed consent to participate in this study.

## Author Contributions

JC and ML: conception, design, and funding acquisition. XD and GL: conception, collection, and assembly of data and project administration. JC, ML, XD, GL, WS, YS, YT, YX, and WL: data analysis and interpretation, manuscript writing, and final approval of manuscript. All authors contributed to the article and approved the submitted version.

## Funding

This study was funded by Foundation of Capital Distinctive Clinical Application Research (Project Number: Z181100001718131).

## Conflict of Interest

The authors declare that the research was conducted in the absence of any commercial or financial relationships that could be construed as a potential conflict of interest.

## Publisher’s Note

All claims expressed in this article are solely those of the authors and do not necessarily represent those of their affiliated organizations, or those of the publisher, the editors and the reviewers. Any product that may be evaluated in this article, or claim that may be made by its manufacturer, is not guaranteed or endorsed by the publisher.
