# Supplementary material for: Sintilimab Combined with Lenvatinib for Advanced Intrahepatic Cholangiocarcinoma in Second-Line Setting—A Multi-Center Observational Study
Source: Front Oncol. 2022 Jul 14;12:907055. doi: 10.3389/fonc.2022.907055 (PMC9333059; doi:10.3389/fonc.2022.907055)
Supplement: Supplementary file 1 [file DataSheet_1.pdf]

stage	surgery	metastas	ID number	first-li	treatment	age	sex
IV		0	228655	AS	Len+PD-1	58	0
IV		0	231743	S-1	Len+PD-1	45	1
IV		0	238160	GP	Len+PD-1	60	1
IV		0	240419	S-1	Len+PD-1	50	1
IV		0	242373	AS	Len+PD-1	58	1
IV		0	246022	GP	Len+PD-1	33	0
II(T2N0M	1	0	247045	S-1	Len+PD-1	47	0
IV	1	1	201722	S-1	Len+PD-1	60	1
II(T2N0M0)		0		S-1	Len+PD-1	67	1
IV		0	245577	S-1	Len+PD-1	44	0
IV	1	1	243158	Gemcitab	Len+PD-1	73	1
IV		0	249483	S-1	Len+PD-1	51	1
	1	1	179197	AS	Len+PD-1	49	1
IV		0	251310	Gemcitab	Len+PD-1	63	1
IV		0	252174	S-1	Len+PD-1	50	1
IV	1	1	229189	GP	Len+PD-1	55	1
II(T2N0M0)		0	214076	GP	Len+PD-1	56	0
IV		0	252462	S-1	Len+PD-1	62	1
IV		0	252891	S-1	Len+PD-1	73	0
	1	1	214299	S-1	Len+PD-1	63	0
IV		0	252476	S-1	Len+PD-1	68	0
IIIB		0	223083	S-1	Len+PD-1	66	0
IV	1	1	249498	S-1	Len+PD-1	66	1
IV		0	223514	S-1	Len+PD-1	54	0
IV		0	216245	S-1	Len+PD-1	62	0
	1	1		S-1	Len+PD-1	72	1
IV	0	0	254399	AS	Len+PD-1	51	0
IIIB	0	0	10772383	Gemcitab	Len+PD-1	52	1
	0	1	10830450	GP	Len+PD-1	67	0
IV	0	0	10863987	S-1	Len+PD-1	59	1
II	0	0	10664859	S-1	Len+PD-1	54	1
	0	1	10878663	GP	Len+PD-1	75	1
	0	1	10797884	GP	Len+PD-1	65	1
II	1	0	10880747	GP	Len+PD-1	72	0
IV	0	0	10703356	GP	Len+PD-1	65	1
IIIB	0	0	10406251	S-1	Len+PD-1	42	0
	0	1	10653780	GP	Len+PD-1	68	0
IV	0	0		S-1	Len+PD-1	48	1
IV	0	0		GP	Len+PD-1	56	1
IV	0	0		S-1	Len+PD-1	68	1
IV	0	0		S-1	Len+PD-1	48	1

ECOG	target o	target o	cirhosis	HBV		DM	lung met	other or
1	15.8	15.2	0	0	0	1	1	liver
1	14.5	15.1	0	1	1	0	0	spleen
1	10.2	6.4	0	1	1	0	1	liver、k
1	8.9	6.1	0	1	1	0	1	bone
1	9.2	12.4	0	1	1	1	0	liver
0	9.1	6.2	0	1	1	0	0	bone
0	5.1	3.2	0	1	1	0	0	0
2	5.6	9.1	1	2	1	1	1	bone
0	8.2	0	0	1	1	1	0	pvtt
1	12.1	2.2	0	0	0	1	0	liver an
1	4.6	6	0	0	0	0	0	0
1	16.2	14.8	0	0	0	0	1	0
1	5.2	1.2	0	1	1	1	0	
1	11.3	9.4	0	1	1	1	1	bone
1	9.1	7.8	0	0	0	0	0	
1	8.6	8.5	0	1	1	1	1	liver
1	6.1	3.8	0	0	0	0	0	0
1	12.9	15.6	1	0	1	1	0	bone
1	13.8	16.8	0	0	0	1	0	
1	4.8	1.6	0	0	0	1	0	0
1	13.2	16.4	0	0	0	0	0	pvtt
1	14.2	13.5	0	0	0	0	0	pvtt
1	6.4	3.8	0	1	1	0	1	liver
1	16.8	20.2	0	0	0	0	0	0
1	12.8	3.2	1	1	1	0	1	0
0	1.5	2.2	1	0	0	0	0	0
1	11.8	12.2	0	0	0	0	0	pvtt and
0	2.8	1.7	1	1	1	1	0	
2	1.5	0.8	0	0	0	0	0	
0	7	3	1	2	1	1	1	
0	6.8	5.7	1	0	0	0	0	
1	2.1	1.6	0	0	0	0	0	
0	2.4	3.3	0	0	0	0	1	
1	8.6	1.8	0	0	0	0	0	
1	6.4	5.3	0	0	0	0	0	bone
0	4.2	1.4	1	1	1	0	0	
1	5.6	2.9	0	0	0	0	0	liver an
1	9.7	12.9	1	1	1	0	1	bone
0	15.6	9.5	0	0	0	0	0	bone，pv
0	6.1	0.5	1	1	1	0	1	pvtt and
0	6.4	7.4	0	0	0	0	0	liver

lymphnod	combined	AFP	CEA	CA19-9	PD-1 (%)	PD-L1 (%	TMB	categori
0	1	4.5	10.2	30.3	0	0	2.2	0
1	1	3.6	1	9.9	1%	0%		
1	1	7.6	23	377.7	18%	15%	6.3	0
1	1	882.59	5.7	188.6	12%	15%		
1	0	3.2	185.5	10245.6	3%	2%	14.8	1
1	1	20.15	1.8	29.4	12%	8%	1.1	0
0	1	2.17	1.7	6.4	10%	0%		
0	0	12.54	15	35.6	2%	0		
0	1	55.19	3.7	13.8	20%	30%		
1	0	0.93	3.3	19.5	50%	70%		
1	0	1.58	7.8	192.2	2%	2%		
1	0	227.43	2.6	246.7	5%	7%		
1	0	5.63	26.3	339.7	12%	15%		
1	1	73.41	2.6	7.4	5%	5%		
1	0	7.05	3.4	643.3	10%	8%	1.2	0
1	0	2.48	21.7	8934.5	0%	0%	2.4	0
0	1	5.8	3.6	276.8	6%	10%	16.8	1
1	0	3.2	2.2	1.8	1%	10%		
1	0	1.94	2.8	62.1	2%	1%		
1	0	1.72	4.1	71.7	25%	30%		
1	1	67251	21.6	151.8	0	0		
1	0	107	50	3326.5	3%	5%		
1	1	1.81	3.3	6.7	5%	20%		
1	1	1.8	47.7	5234.7	1%	2%		
0	1	2.1	3.6	859	10%	20%		
1	0	3.6	4.5	824.2	2%	1%		
1	0	3.98	1.3	21.9	5%	6%	2.6	0
1	2	16.95	1.85	152.82	0.25	0.3		
1	0	6.35	1.55	1.3	0.2	0.2		
1	2	10.99	2.88	12.67	0.12	0.15	6.38	
0	2	4.48	5.26	62677	1	0		
1	2	3.16	3.21	108	0.06	0.08		
1	0	2.1	21.69	2	0	0	1	
0	1	2.32	47	18.67	0.15	0.18		
0	2	7.2	3.68	41.65	0.05	0.05	6.4	
1	2	4.79	1.04	4.11	0.03	0.05	6.67	
1	0	3.63	10.31	42.77	0.05	0.03	11.96	
1	1	18.9	5.08	3258.9	0%	0%		
1	0	3.34	4.43	82.9	18%	20%	3.4	
1	1	1.93	2.64	27.2	28%	25%	7.53	
1	1	1.81	1.95	67.6	5%	8%		

efficacy	NLR	categori	PLR	categori	NEUT	LY	WBC	platelet
SD	1.9	0	143.9	0	3.29	1.73	5.26	249
SD	4.48	1	186.9	1	5.47	1.22	7.85	228
PR	3.47	0	99.5	0	7.42	2.14	11.75	213
PR	1.85	0	61.18	0	4.71	2.55	8.06	156
PD	2.8	0	139.1	0	6.29	2.25	9.86	313
PR	5	1	226	1	6.15	1.23	7.95	278
PR	1.95	0	118.9	0	2.57	1.32	4.31	157
PD	3.18	0	122.58	0	1.97	0.62	3.05	76
PR	3.18	0	159.5	0	3.85	1.21	5.58	193
PR	4.57	1	82.57	0	4.98	1.09	7.19	90
PD	2.02	0	82.57	0	4.4	2.18	7.53	180
SD	4.3	1	81.71	0	7.05	1.64	9.42	134
PR	4.78	1	280	1	4.06	0.85	5.72	238
SD	4.96	1	172.57	0	5.61	1.13	7.53	195
SD	2.23	0	115.4	0	5.35	2.4	9.14	277
SD	1.22	0	46.82	0	2.68	2.2	5.25	103
PR	1.86	0	114.05	0	3.45	1.85	5.63	211
PD	8.47	1	217.19	1	10.84	1.28	13.4	278
PD	1.6	0	72	0	3.02	1.89	5.61	136
PR	2.43	0	113.95	0	4.18	1.72	6.38	196
PD	3.31	0	107.69	0	4.3	1.3	6.35	140
SD	1.63	0	97.79	0	6.62	4.07	11.73	398
PR	3.03	0	159.57	0	2.85	0.94	4.56	150
PD	4.6	1	248.29	1	9.42	2.05	12.27	509
PR	5.41	1	254.9	1	2.76	0.51	3.98	130
PD	6.25	1	170.15	0	4.19	0.67	5.34	114
SD	1.73	0	135.45	0	1.9	1.1	3.4	149
PR	2.82	0	108.1	0	5.96	2.11	9.25	228
PR	1.63	0	81.6	0	3.35	2.06	6.93	168
PR	3.35	0	90.5	0	3.18	0.95	4.68	86
SD	2.67	0	179.2	0	5.89	2.21	8.82	396
SD	3.08	0	182	0	4.1	1.33	6.84	242
PD	2.25	0	142.4	0	2.81	1.25	4.36	178
PR	4.2	1	203.2	1	3.91	0.93	5.39	189
SD	2.87	0	134.2	0	5.54	1.93	8.37	259
PR	2.18	0	96.4	0	1.81	0.83	2.95	80
PR	2.29	0	122.7	0	3.44	1.5	5.37	184
PD	3.57	0	191.6	0	7.75	2.03	11.38	389
PR	5.95	1	191.6	0	6.96	1.17	8.99	282
PR	2.68	0	146.2	0	5.22	1.95	8.09	285
SD	4.05	1	263.4	1	4.98	1.23	6.76	324

TTP	progress	OS	death
6.9	1	26.2	1
4.5	1	8.5	1
6.5	1	16.6	1
7.6	1	11.2	1
2.2	1	6.9	1
8.4	1	12.1	0
11.2	0	11.2	0
1.8	1	5.8	1
14.1	0	14	0
14.4	0	14.4	0
3	1	9	1
2.8	1	4.1	1
11.6	1	16.2	0
4.5	0	4.5	0
3.7	0	3.7	0
3.8	1	9.6	1
7.1	0	7.1	0
1.8	1	3.2	1
2.4	1	4.2	0
3.4	0	3.4	0
2.3	1	3.6	0
2.9	1	4.2	1
3.2	0	3.2	0
1.5	1	3.6	1
18.8	1	21.9	1
1.8	1	4.2	0
5.5	1	6.6	0
6.6	1	9.3	1
16.9	1	27	0
6	0	6	0
9.9	1	17	0
7	0	7	0
3	1	15	0
6	0	6	0
13.8	1	16	0
14	1	40	1
6.2	1	9	0
2.2	1	6.9	1
5.8	0	5.8	0
8	0	8	0
4.1	1	6.2	0
